# Evaluation of DNAmAge in paired fresh, frozen, and formalin-fixed paraffin-embedded heart tissues

**DOI:** 10.1371/journal.pone.0299557

**Published:** 2024-05-08

**Authors:** Paulina Pruszkowska-Przybylska, Mikkel Eriksen Dupont, Stine Bøttcher Jacobsen, Morten Smerup, Jacob Tfelt-Hansen, Niels Morling, Jeppe Dyrberg Andersen

**Affiliations:** 1 Department of Anthropology, Faculty of Biology and Environmental Protection, University of Łódź, Łódź, Poland; 2 Section of Forensic Genetics, Department of Forensic Medicine, Faculty of Health and Medical Sciences, University of Copenhagen, Copenhagen, Denmark; 3 Department of Cardiothoracic Surgery, Rigshospitalet, Copenhagen University Hospital, Copenhagen, Denmark; 4 Department of Cardiology, Rigshospitalet, Copenhagen University Hospital, Copenhagen, Denmark; Universita degli Studi di Salerno, ITALY

## Abstract

The continued development in methylome analysis has enabled a more precise assessment of DNA methylation, but treatment of target tissue prior to analysis may affect DNA analysis. Prediction of age based on methylation levels in the genome (DNAmAge) has gained much interest in disease predisposition (biological age estimation), but also in chronological donor age estimation in crime case samples. Various epigenetic clocks were designed to predict the age. However, it remains unknown how the storage of the tissues affects the DNAmAge estimation. In this study, we investigated the storage method impact of DNAmAge by the comparing the DNAmAge of the two commonly used storage methods, freezing and formalin-fixation and paraffin-embedding (FFPE) to DNAmAge of fresh tissue. This was carried out by comparing paired heart tissue samples of fresh tissue, samples stored by freezing and FFPE to chronological age and whole blood samples from the same individuals. Illumina EPIC beadchip array was used for methylation analysis and the DNAmAge was evaluated with the following epigenetic clocks: Horvath, Hannum, Levine, Horvath skin+blood clock (Horvath2), PedBE, Wu, BLUP, EN, and TL. We observed differences in DNAmAge among the storage conditions. FFPE samples showed a lower DNAmAge compared to that of frozen and fresh samples. Additionally, the DNAmAge of the heart tissue was lower than that of the whole blood and the chronological age. This highlights caution when evaluating DNAmAge for FFPE samples as the results were underestimated compared with fresh and frozen tissue samples. Furthermore, the study also emphasizes the need for a DNAmAge model based on heart tissue samples for an accurate age estimation.

## 1 Introduction

The correct storage of a sample is pivotal in forensic [1] and medical sciences [2, 3]. Different storage conditions for tissues may reflect different purposes for tissue analyses, but usually a storage condition is selected to avoid tissue degradation [4]. A commonly used method for sample storage is freezing, which requires large freezing capacities if the sample archive is large. Another commonly used tissue preservation method is formalin fixation and paraffin embedding (FFPE) [5]. The advantage of the FFPE storage is that it is stored at room temperature and is relatively cost-efficient. FFPE of tissues is typically carried out where histopathological investigations are a part of the subsequent analysis as seen in forensic and medical investigations [2, 6]. The different types of storage conditions may have potential impact on DNA quality as well as on DNA methylation levels [4, 7]. Bulla et al. [8] evaluated the temperature impact of DNA methylation levels in blood samples and showed that higher/lower temperature had a significant impact on the DNA extraction yield, but not on DNA integrity or methylation.

The potential role of epigenetics in forensic investigation lies in tissue identification of trace sample and age estimation of donor and has been described previously [9, 10]. Prediction of age based on methylation levels in tissue is known as DNAmAge [11, 12]. The DNAmAge may be calculated using various prediction tools and was originally proposed by Horvath in 2013 [13]. Currently, there are numerous models (epigenetic clocks) for DNAmAge, including Hannum [14], Levine [15], Horvath skin+blood clock (Horvath2) [16], PedBE [17], Wu [18], BLUP [19], EN [19] and TL [20] (Table 1), which were developed for various tissues and age groups. The epigenetic clocks are used in various studies related with chronological age estimation [21], but also with aging process, lifestyle, and disease predisposition (biological age estimation) [22–24].

**Table 1:** Characteristic of the different epigenetic clocks.

Name of the clock	Type of tissue	Target group	Number of used CpGs	Number of missing CpGs	Missing CpGs
Horvath’s clock [13]	Pan-tissue	No defined	353	19	cg02654291 cg02972551 cg06144905 cg09785172 cg09869858 cg13682722 cg14329157 cg16494477 cg17408647 cg19273182 cg19945840 cg27319898 cg04431054 cg05590257 cg06117855 cg19046959 cg19569684 cg24471894 cg27016307
Hannum’s clock [14]	Blood	No defined	71	8	ch.2.30415474Fcg24079702 cg14361627 cg07927379 cg18473521 ch.13.39564907Rcg09651136 cg21139312
Levine (PgenoAge-mortality predictor) [15]	Blood	No defined	513	3	cg26665419 cg06144905 cg08212685
Horvath’s skin+blood clock (Horvath2) [16]	Skin and blood	No defined	391	6	cg10959651 cg18303397 cg21874213 cg09183146 cg06144905 cg21944491
PedBE clock [17]	Buccal epithelial swabs	Individuals aged 0–20	84	1	cg06144905
Wu clock [18]	Blood	Children	111	4	cg05352668 cg08724636 cg19162158 cg24471894
BLUP clock [19]	Blood and saliva	No defined	319607	4208	Too numerous data
EN clock [19]	Blood and saliva	No defined	514	2	cg18768612 cg05694771
TL clock (telomer length predictor) [20]	Blood	No defined	140	3	cg27432653 cg08276993 cg21874213

Most of the tools for biological age estimations are focused on DNA methylation [25]. To receive reliable results, it is crucial to have proper storage of the samples to avoid changes in the DNA integrity or DNA methylation. Additionally, one of the challenges in chronological and biological age estimation is to develop tissue and disease-specific clocks [26, 27]. Nevertheless, the accuracy of DNAmAge prediction is improving, and methylation data is now available for many different tissues [4], but the available data for heart tissue is still limited. One study, by Pavanello et al. [28], investigated DNAmAge in heart tissue and showed that DNAmAge of the heart tissue was lower than the chronological age.

The importance of studying the DNAmAge in the various human tissues is needed in forensic practice to properly distinguish the tissue type and age of the investigated individuals from the crime scene [29]. Moreover, various cardiac diseases have been linked with DNA methylation profile that may predict future recurrence or complication [30]. The importance of investigated the heart tissue stems from the tissue structure which is composed of different cell types (such as. including cardiomyocytes, endothelial cells, fibroblasts, smooth muscle cells, inflammatory and microvascular cells, and a small pool of pluripotent stem cells [31]. In contrast to other tissues the heart tissue has a minimal regenerative capacity [32, 33]. However, adult heart tissue presents little changes in the methylome [34]. For these reasons, the current study shows new insights that extend existing knowledge about heart tissue stored under different conditions.

The aim of this study was to evaluate DNAmAge in paired fresh, frozen, and FFPE heart tissues using different epigenetic clocks and compare it to that of whole blood samples and chronological age from the same individuals.

To the best of our knowledge, there have been no investigations comparing the DNAmAge of untreated fresh tissue with tissues stored by freezing or FFPE. Furthermore, studies in DNAmAge of heart tissue is very limited and increasing knowledge of DNAmAge estimation in various tissues is important to better understand how these may be used in biological age estimation and disease predisposition.

## 2 Material and methods

### 2.1 Ethics

The study was approved by the Committees of Health Research Ethics in the Capital Region of Denmark (H-20039524). The study is registered in the University of Copenhagen’s record of research projects, including personal data (514-0528/20-3000), and it complies with the rules of the General Data Protection Regulation (Regulation (EU) 2016/679). Informed written consent was collected from all individuals. Patient data were pseudonymised.

### 2.2 Study population and tissue collection

Human right atrial appendage (RAA) tissue was collected from 10 individuals: 9 males and 1 female (mean age: 66.5, SD = 9.1) that underwent scheduled cardiac surgery at Rigshospitalet, Copenhagen, Denmark between 02-23/06/2021. All samples were divided into three pieces: one piece was used for DNA extraction immediately after tissue collection (fresh tissue), one was frozen at -80°C (frozen tissue), and the last piece was fixed in formalin and embedded in paraffin (FFPE tissue).

A blood sample was collected from each patient the day before the scheduled cardiac surgery. A total of 4 ml whole blood was collected in EDTA coated tubes. Whole blood samples were stored at -20°C for 14 months prior to DNA extraction. The DNA was extracted immediately from fresh samples and then further procedures were performed within 2 months. Frozen samples were instantly placed in the –80°C, DNA extraction and methylation analyses were performed within three months from the collection date. The FFPE samples were prepared on the date of sample collection and further steps took place within the following 4 months. The detailed timeline can be found in S1 Data.

### 2.3. Laboratory work

Laboratory work was conducted according to Infnium® HD Assay Methylation Protocol Guide (2015) [35].

#### 2.3.1 Formalin-fixed and paraffin-embedded samples

RAA tissue samples were fixed with 4% buffered formaldehyde using the Biopsafe Biopsy Sample System (BiopSafe, Denmark). Fixation times ranged from 23–97 hours (median: 62 hours). Tissues were dehydrated and paraffin treated using a Tissue-Tek VIP 6 AI (Sakura Finetek Europe, the Netherlands) and included the following incubations: 1 x 4% buffered formaldehyde for 60 min., 6 x EtOH for 90 min. with increasing concentrations of EtOH, 2 x Histolab Clear (Histolab Products AB, Sweden) for 60 min., 1 x Histolab Clear for 120 min., and 4 x Paraffin for 80 min. Lastly, tissues were embedded in paraffin.

#### 2.3.2 DNA extraction

DNA from fresh and frozen RAA tissue was extracted using DNeasy Blood & Tissue Kit (Qiagen, Germany) following the manufacturers recommendations. 3 mm cubes were used as input, and the tissues were homogenised for 5 x 2 min at 20 Hz using the TissueLyser II (Qiagen, Germany). To ensure complete digestion of proteins, 40 μl proteinase K was added. DNA was eluted in 100 μL elution buffer.

DNA from FFPE tissue was extracted using QIAamp® DNA FFPE Tissue kit (Qiagen, Germany) following the manufacturers recommendations with the exception that Proteinase K digestion was conducted overnight until the tissue was completely dissolved. A total of 5 x 20 μm slides (tissue ~ 7 x 7 mm) were used as input per extraction. Paraffin was removed by twice using xylene followed by a wash in 96–100% ethanol. DNA was eluted in 55 μL elution buffer.

DNA extractions from nine of the ten patients were performed in duplicates. A single DNA extraction from each storage condition was performed from individual five, due to limited amount of tissue. The quantity of the DNA was measured using the qubit dsDNA HS assay kit (Invitrogen, USA). The quality of the DNA was evaluated using the Infinium HD FFPE QC kit (Illumina, USA) and the Quantifiler Trio® DNA Quantification kit (Thermo Fisher Scientific, USA) following the manufacturer’s protocol. Both qPCR methods were performed using the Applied Biosystems 7500 Real-Time PCR System (Thermo Fisher Scientific, USA).

#### 2.3.3 DNA methylation array

Bisulfite conversion was performed using the EZ DNA Methylation kit (Zymo Research Corporation, USA) following the manufacturer’s protocol with 175–250 ng DNA as input. The converted DNA was eluted in 10 μL elution buffer. DNA extracted from FFPE tissue was restored using the Infinium HD FFPE DNA Restore kit (Illumina, USA) following manufactures protocol. The levels of DNA methylation were quantified using the Infinium MethylationEPIC v1.0 BeadChip Kit (Illumina, USA) following the manufacturer’s protocol. The prepared slides were scanned using the iScan System (Illumina, USA).

### 2.4 DNAmAge

For the methylation analysis, the R (version 4.2.2) package–*Rnbeads* was used [36]. In the *Rnbeads* procedures, quality control and normalisation were implemented. During quality control, some of the samples and probes were excluded if “genetic noise” was detected. These deviations verified by applied algorithm can indicate technical problems of the microarray-based analysis, contamination with DNA samples from other individuals, or deviations from the diploid case (e.g., aneuploid cancer samples) [36]. The *bmiq* normalisation was used for all DNAmAge analysis. As an outcome of the filtering and quality control procedures, 2969 probes and one sample were excluded (66 samples and 842,052 probes per sample were retained). Beta values were extracted for the downstream analysis and the Bioconductor package *methylclock* was used to predict DNAmAge [37] using the following epigenetic clocks: Horvath, Hannum, Levine (PhenoAge), Horvath skin+blood clock (Horvath2), PedBE, Wu, BLUP, EN, and TL (Table 1).

For each individual, the mean of the two replicates was used in the downstream analysis.

Some of the CpGs used in the proposed clocks were not present on the EPIC platform and the list of missing CpGs island for each epigenetic clock is shown in the Table 1.

To evaluate the clock which shows the lowest differences between storage conditions there were calculated delta values of epigenetic age for each methylClock according to equations: Delta 1 = frozen-FFPE, Delta 2 = fresh-FFPE, Delta 3 = fresh-frozen.

The results were presented in Table 3.

### 2.5 Statistics

We evaluated differences in the DNAmAge among the three different types of storage, whole blood, and chronological age. Friedman Test for dependent samples was used. For a post-hoc test, the pairwise Wilcoxon rank sum test with a Bonferroni correction was applied. All analyses were performed in R (version 4.2.2).

## 3 Results

This study compared epigenetic age (DNAmAge) in paired fresh, frozen, and FFPE heart tissues using nine different epigenetic clocks (Table 1) and compared it to that of whole blood samples and chronological age from the same individuals.

### 3.1 Differences in DNAmAge of heart tissue stored under different conditions

**Fig 1:**
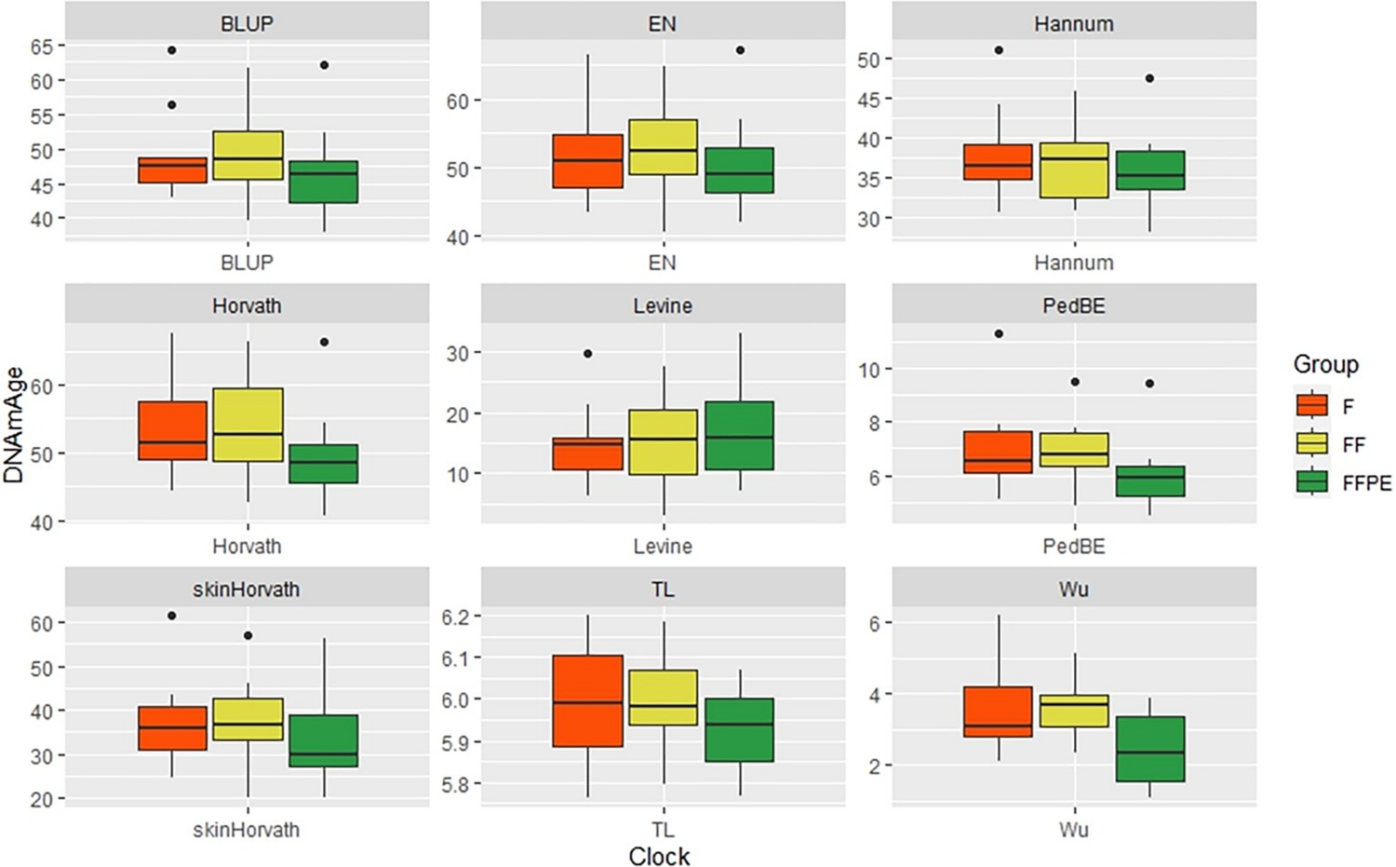
Differences in DNAmAge of heart tissue stored under different conditions and comparison to DNAmAge in whole blood and chronological age. Friedman Test for dependent samples: Horvath (chi-squared = 28.36, df = 4, p<0.001), post hoc tests: F&B (p = 0.002), FF&B(p = 0.002), FFPE&B(p<0.001), FFPE&CH (p = 0.005); Hanum (chi-squared = 29.42, df = 4, p<0.001), Levine (chi-squared = 27.64, df = 4, p<0.001), skinHorvath (chi-squared = 28.09, df = 4, p<0.001), BLUP (chi-squared = 28.36, df = 4, p<0.001) and EN (chi-squared = 25.11, df = 4, p<0.001), F&B (p<0.001), FF&B (p<0.001), FFPE&B (p<0.001), F&CH (p<0.001), FF&CH (p<0.001), FFPE&CH (p<0.001); PedBE (chi-squared = 31.56, df = 4, p<0.001) and Wu (chi-squared = 33.16, df = 4, p<0.001), TL (chi-squared = 29.60, df = 4, p<0.001) post hoc: F&B (p<0.001), FF&B (p<0.001), FFPE&B (p<0.001), F&CH (p<0.001), FF&CH (p<0.001), FFPE&CH (p<0.001), F&B (p<0.001) (S2 Data). The Friedman test for dependent samples with post-hoc Wilcoxon rank sum exact test and a Bonferroni correction showed statistically significant lower DNAmAge for fresh, fresh frozen and FFPE compared to the chronological age for the five methylclocks: Hanum, Levine, Horvath skin+blood clock (Horvath2), PedBE and Wu. No statistically significantly difference was observed between DNAmAge for blood and chronological age. We found Hanum, and Levine predicted DNAmAge to be statistically significant lower in fresh, frozen, and FFPE compared with DNAmAge of blood and chronological age. We found Hovarth 2 to have statistically significant lower DNAmAge compared with that of blood, and DNAmAge of PedBE and Wu in blood and fresh frozen to be statistically significantly lower than that of chronological age. b) Differences in DNAmAge of heart tissue stored under different conditions and comparison to DNAmAge and chronological age. Friedman Test for dependent samples: Horvath (chi-squared = 28.36, df = 4, p<0.001), Hanum (chi-squared = 29.42, df = 4, p<0.001), Levine (chi-squared = 27.64, df = 4, p<0.001), skinHorvath (chi-squared = 28.09, df = 4, p<0.001), BLUP (chi-squared = 28.36, df = 4, p<0.001) and EN (chi-squared = 25.11, df = 4, p<0.001), PedBE (chi-squared = 31.56, df = 4, p<0.001) and Wu (chi-squared = 33.16, df = 4, p<0.001), TL (chi-squared = 29.60, df = 4, p<0.001) post hoc: F&CH (p<0.001), FF&CH (p<0.001), FFPE&CH (p<0.001) (S2 Data). A second Friedman test for dependent samples with post-hoc Wilcoxon rank sum exact test and a Bonferroni correction was carried out among storage conditions and chronological age, but this time without blood. The test showed that DNAmAge was statistically significant lower for all three storage conditions compared to the chronological age for all test epigenetic clocks. c) Differences in DNAmAge of heart tissue stored under different conditions. The pairwise Wilcoxon rank sum test with a Bonferroni correction: not statistically significant (S2 Data). A third Friedman test for dependent samples with post-hoc Wilcoxon rank sum exact test and a Bonferroni correction was carried out to test for statistical significance among the DNAmAge for three different storage conditions. PedBE, Wu, and BLUP showed statistically significant difference. After applying post-hoc pairwise Wilcoxon rank sum test with a Bonferroni correction, none of the epigenetic clocks showed statistically significant differences among the three storage conditions.

We found statistically significant differences in DNAmAge among storage conditions for (Hanum, Levine, Horvath skin+blood clock (Horvath2), PedBE and Wu). Samples stored as FFPE had a lower DNAmAge compared to that of fresh and frozen tissue samples (Fig 1 and Table 2).

**Table 2:** Descriptive statistics and differences between three types of storage evaluating by different epigenetic clocks.

Clock	Group	N	Means*	SD	Q1	Median	Q3
Horvath	FFPE	10	49.46	7.13	45.49	48.46	51.53
	FF	10	54.10	7.77	48.43	52.62	60.33
	F	10	53.70	7.67	48.46	51.54	59.04
	B	9	72.50	6.47	70.99	71.89	72.86
Hannum	FFPE	10	35.86	5.41	33.43	35.13	38.65
	FF	10	37.22	5.28	31.70	37.26	39.89
	F	10	37.88	5.94	34.54	36.39	39.91
	B	9	72.50	6.47	70.99	71.89	72.86
Levine	FFPE	10	16.59	8.32	10.62	15.68	21.99
	FF	10	15.07	7.63	9.87	15.67	21.72
	F	10	14.93	6.63	10.54	14.63	16.09
	B	9	57.49	6.44	52.35	59.00	60.30
skinHorvath	FFPE	10	33.37	10.35	27.39	29.98	40.71
	FF	10	37.97	9.90	33.06	36.55	42.97
	F	10	37.25	10.51	30.01	35.83	40.83
	B	9	66.65	7.91	63.27	65.73	72.76
PedBE	FFPE	10	6.02	1.38	5.18	5.90	6.45
	FF	10	6.92	1.29	6.29	6.77	7.62
	F	10	6.98	1.80	6.09	6.50	7.77
	B	9	12.64	1.57	11.51	13.05	13.36
Wu	FFPE	10	2.45	1.05	1.49	2.35	3.36
	FF	10	3.64	0.93	2.92	3.71	4.01
	F	10	3.51	1.24	2.79	3.07	4.54
	B	9	13.04	0.76	12.55	12.85	13.53
TL	FFPE	10	5.93	0.10	5.83	5.94	6.01
	FF	10	5.99	0.11	5.93	5.98	6.09
	F	10	5.99	0.15	5.88	5.99	6.12
	B	9	6.51	0.22	6.38	6.58	6.67
BLUP	FFPE	10	46.79	6.76	41.78	46.43	48.42
	FF	10	49.60	6.42	45.26	48.47	52.55
	F	10	49.06	6.55	44.53	47.61	48.77
	B	9	67.82	9.74	62.43	69.55	72.67
EN	FFPE	10	50.60	7.35	45.39	49.03	53.88
	FF	10	52.94	7.35	48.82	52.36	57.23
	F	10	52.00	7.12	46.70	50.92	55.10
	B	9	64.66	9.51	59.39	65.85	70.96
	CH	10	66.50	9.56	60.00	66.50	75.00

FFPE-formalin-fixed, paraffin-embedded; FF-frozen; F-fresh; B-blood, CH- chronological age

*means value for all individuals

The mean delta values among DNAmAge for fresh, frozen and FFPE samples were calculated as presented in the Table 3. Mean delta values showed that FFPE samples were smaller than fresh and frozen samples for all methylclocks besides Levine clock. We found the smallest non-significant differences among FFPE, fresh, and frozen for the Levine (PhenoAge) epigenetic clock (Table 3, S1 Fig).

**Table 3:** Delta means values for various methylclocks in different types of storage.

Clock	Delta FF-FFPE	Delta F-FFPE	Delta F-FF
**Horvath** *	4.64	4.24	-0.40
**Hannum** *	1.36	2.02	0.66
**Levine** *	-1.52	-1.66	-0.14
**skinHorvath**	4.59	3.88	-0.72
**PedBE**	0.90	0.96	0.06
**Wu**	1.19	1.06	-0.13
**TL**	0.07	0.06	0.00
**BLUP**	2.80	2.27	-0.53
**EN**	2.34	1.40	-0.94

*statistically significant

### 3.2 Differences in DNAmAge of heart tissue and whole blood samples

The heart tissue samples showed lower DNAmAge than whole blood regardless of storage methods for all the evaluated epigenetic clocks (Fig 1 and Table 2).

## 4 Discussion

In this study, we evaluated DNAmAge in paired fresh, frozen, and FFPE heart tissues using nine different epigenetic clocks. We show for the first time how the DNAmAge of different epigenetic clock are affected by freezing and FFPE storage methods by comparing results to untreated fresh material. DNAmAge of FFPE heart was found to be lower than those obtained from fresh and frozen tissues for all epigenetic clocks investigated.

It is known that analysis of DNA purified from FFPE is feasible, but the quantity and quality of the DNA is lower compared to that of frozen tissue, thus it was suggested previously by Chung et al. (2008) [38] in their RNA study to standardise the results when the FFPE samples were used. Other studies indicated that FFPE preparation did not affect DNA quality nor quantity [5]. However, there is more studies which indicated that FFPE affected DNA methylation [39, 40]. It is widely known that formalin fixation causes crosslinking of proteins with DNA [41], and results in fragmentation of the DNA when the crosslinking is broken [42]. The formalin effect on methylation levels has only been vaguely investigated [43, 44]. So far, there has been no evaluation on how FFPE sample preparation influences the DNA methylation in heart tissue and how it affects CpGs which are included in epigenetic clocks. We obtained different epigenetic age for some of the clocks in blood and heart samples: fresh and FFPE as well, thus it may indicate different methylation level. These results are opposite to, Moran et al. (2014) [45] who did not find differences between DNA methylation in frozen and FFPE samples which came from the various tissues: breast, colon, kidney, lung, ovary, pancreas, prostate, stomach.

Another issue is temperature storage. Bulla et al. (2016) [8] evaluated the impact of different storage temperatures on DNA methylation from blood samples and showed that temperature of the storage has a significant impact on the DNA extraction yield, but not on DNA integrity or methylation. We observed that there were some differences between epigenetic age of fresh and frozen heart tissue thus may be related to some differences in DNA methylation.

The storage time of FFPE samples is another factor that could influence DNA methylation because FFPE samples are exposed to oxidation [46]. Watanabe et al. (2017) [47] showed that storage time may limit the amount of available DNA in FFPE tissue, however they did not measure the effect on DNA methylation. In our investigation, the FFPE samples were stored one month longer than frozen samples before DNA extraction, thus the time difference is relatively small, and we do not suspect this to influence the results of this study. Additionally, in blood samples, Li et al. (2018) [48] presented that 20 years of storage of FF samples did not affect DNA methylation.

In 2020, Pavanello et al. [28] applied the model proposed by Zbieć-Piekarska et al. [49] with five CpGs in *ELOVL2*, C1orf132, *TRIM59*, *KLF14*, and *FHL2*, and a standard error of the estimate of 4.5 years on heart tissue. Like many other models [13–16, 18–20], the model proposed by Zbieć-Piekarska et al. [49] was developed on whole blood. However, other common epigenetic clocks seem to be more precise for example Horvath’s clock (2013) [13] that predicts age with a median error of 3.6 years across a wide array of diverse tissues and cells. For this reason, we predicted DNAmAge with many different epigenetic clocks and we confirmed that DNAmAge in heart tissue was lower compared to DNAmAge in whole blood samples independently on epigenetic clock.

In 2023 Mongelli et al. [50] proposed the first algorithm that fit to heart tissue among patients undergoing cardiac surgery for an aortic valvular replacement (AVR) or coronary artery bypass graft (CABG). However, this algorithm needs verification and wider investigation compering with healthy population to able interpopulation comparisons. Consequently, our study underlines the needs to develop a new epigenetic clock specific for heart tissue.

Moreover, we underlined that, the Levine clock (PhenoAge) showed the smallest differences in DNAmAge among F, FF, and FFPE. Levine et al. (2018) [15] included, besides CpGs associated with chronological age, also CpGs associated with phenotypic traits and mortality rate indicators, including body mass index (BMI), physical activity, alcohol consumption, smoking, and mental health. Our results indicate that this clock is the least sensitive to FFPE treatment, however, more studies are needed to evaluate Levine’s clock in other FFPE tissues to find out if the DNAmAge shows similar performance as heart tissue.

This study has potential application in medical and forensic cases in which age determination is needed of heart tissue and FFPE samples. The investigation on the bigger group may add more statistical power and allows to develop the universal adjustment for different storage, which can be apply in methylation analysis for DNAmAge.

## 5 Conclusions

We used nine different epigenetic clocks and found that FFPE affected DNAmAge more than freezing of the samples, as the difference between DNAmAge of FFPE and fresh tissue was larger compared to DNAmAge of fresh and frozen tissues. Furthermore, the DNAmAge of cardiac tissues was found to be lower than that of whole blood and chronological age. This highlights the need for the development of a heart tissue based DNAmAge model for more accurate results.

## 6 Limitations

Beside study has potential application in medical and forensic cases in which age determination is needed of heart tissue and FFPE samples. The study has however some limitations with the relatively small number of paired samples used. More numerous study group would strengthen the results. Nevertheless, our results underline awareness of methylation changes depending on type of the storage.

One of the samples was not run in the duplicates that might influence the results.

Considering applied epigenetic clocks there were some discrepancies in included CpGs and some of them were missing that might provide unauthenticated results.

## Supporting information

S1 Fig Differences in DNmAge among FFPE, fresh, and frozen samples. (JPEG)

S1 Data Detailed statistics. (XLSX)

S2 Data Detailed information about dates and samples preparation. (DOCX)

S3 Data File with the results of the DNAmAge for each clock. (XLSX)
