# Supplementary figures and images for: Evaluation of DNAmAge in paired fresh, frozen, and formalin-fixed paraffin-embedded heart tissues
Source: PLoS One. 2024 May 8;19(5):e0299557. doi: 10.1371/journal.pone.0299557 (PMC11078437; doi:10.1371/journal.pone.0299557)

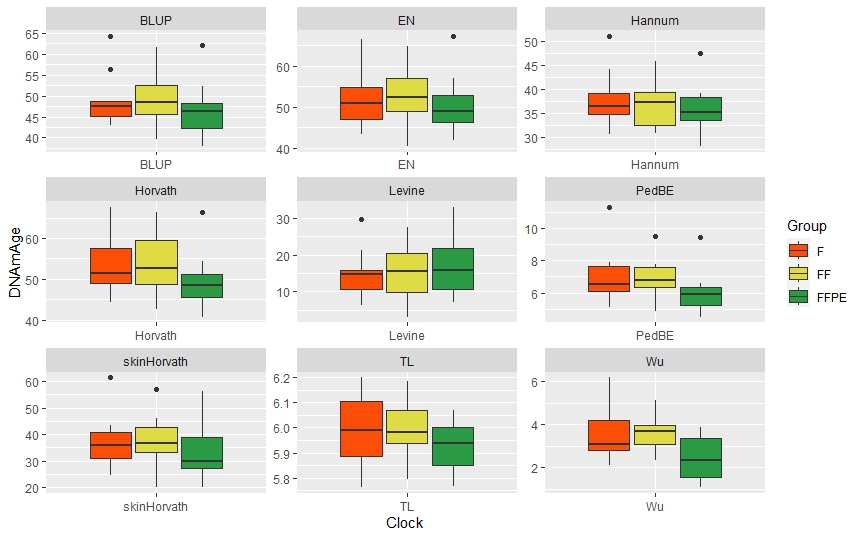

Supplement: S1 Fig — (JPEG) [file pone.0299557.s001.jpeg]
