# Supplementary material for: Evaluation of DNAmAge in paired fresh, frozen, and formalin-fixed paraffin-embedded heart tissues
Source: PLoS One. 2024 May 8;19(5):e0299557. doi: 10.1371/journal.pone.0299557 (PMC11078437; doi:10.1371/journal.pone.0299557)
Supplement: S2 Data — (DOCX) [file pone.0299557.s003.docx]

**Supplementary Data 2**

1. **Differences in DNAmAge of heart tissue stored under different conditions and comparison to DNAmAge in whole blood and chronological age.**

**Horvath**

Friedman chi-squared = 28.356, df = 4, p-value = 1.057e-05

Pairwise comparisons using Wilcoxon rank sum exact test

P value adjustment method: Bonferronie

B CH F FF

CH 1.00000 - - -

F 0.00152 0.08931 - -

FF 0.00152 0.08931 1.00000 -

FFPE 0.00087 0.00487 1.00000 1.00000

**Hannum**

Friedman chi-squared = 29.422, df = 4, p-value = 6.416e-06

Pairwise comparisons using Wilcoxon rank sum exact test

B CH F FF

CH 1.00000 - - -

F 0.00043 0.00022 - -

FF 0.00022 0.00011 1.00000 -

FFPE 0.00022 0.00011 1.00000 1.00000

**Levine**

Friedman chi-squared = 27.644, df = 4, p-value = 1.472e-05

Pairwise comparisons using Wilcoxon rank sum exact test

B CH F FF

CH 0.34987 - - -

F 0.00022 0.00011 - -

FF 0.00022 0.00011 1.00000 -

FFPE 0.00022 0.00011 1.00000 1.00000

**skinHorvath**

Friedman chi-squared = 28.089, df = 4, p-value = 1.197e-05

Pairwise comparisons using Wilcoxon rank sum exact test

B CH F FF

CH 1.00000 - - -

F 0.00087 0.00076 - -

FF 0.00043 0.00043 1.00000 -

FFPE 0.00043 0.00043 1.00000 1.00000

**PedBE**

Friedman chi-squared = 31.556, df = 4, p-value = 2.358e-06

Pairwise comparisons using Wilcoxon rank sum exact test

B CH F FF

CH 0.00022 - - -

F 0.00087 0.00011 - -

FF 0.00022 0.00011 1.00000 -

FFPE 0.00022 0.00011 1.00000 0.63013

**Wu**

Friedman chi-squared = 33.156, df = 4, p-value = 1.11e-06

Pairwise comparisons using Wilcoxon rank sum exact test

B CH F FF

CH 0.00022 - - -

F 0.00022 0.00011 - -

FF 0.00022 0.00011 1.00000 -

FFPE 0.00022 0.00011 1.00000 0.23231

**TL**

Friedman chi-squared = 29.6, df = 4, p-value = 5.903e-06

Pairwise comparisons using Wilcoxon rank sum exact test

B CH F FF

CH 0.00022 - - -

F 0.00043 0.00011 - -

FF 0.00022 0.00011 1.00000 -

FFPE 0.00022 0.00011 1.00000 1.00000

**BLUP**

Friedman chi-squared = 29.067, df = 4, p-value = 7.577e-06

Pairwise comparisons using Wilcoxon rank sum exact test

B CH F FF

CH 1.00000 - - -

F 0.00411 0.00325 - -

FF 0.00260 0.00487 1.00000 -

FFPE 0.00087 0.00130 1.00000 1.00000

**EN**

Friedman chi-squared = 25.511, df = 4, p-value = 3.97e-05

Pairwise comparisons using Wilcoxon rank sum exact test

B CH F FF

CH 1.00000 - - -

F 0.00411 0.00325 - -

FF 0.00260 0.00487 1.00000 -

FFPE 0.00087 0.00130 1.00000 1.00000

**2. Differences in DNAmAge of heart tissue stored under different conditions and comparison to DNAmAge and chronological age.**

**Horvath**

Friedman chi-squared = 19.32, df = 3, p-value = 0.0002348

Pairwise comparisons using Wilcoxon rank sum exact test

CH F FF

F 0.0536 - -

FF 0.0536 1.0000 -

FFPE 0.0029 0.9930 0.9930

**Hannum**

Friedman chi-squared = 19.44, df = 3, p-value = 0.0002217

Pairwise comparisons using Wilcoxon rank sum exact test

CH F FF

F 0.00013 - -

FF 6.5e-05 1.00000 -

FFPE 6.5e-05 1.00000 1.00000

**Levine**

Friedman chi-squared = 18.48, df = 3, p-value = 0.0003501

Pairwise comparisons using Wilcoxon rank sum exact test

CH F FF

F 6.5e-05 - -

FF 6.5e-05 1 -

FFPE 6.5e-05 1 1

**SkinHorvath**

Friedman chi-squared = 20.28, df = 3, p-value = 0.0001485

Pairwise comparisons using Wilcoxon rank sum exact test

CH F FF

F 0.00045 - -

FF 0.00026 1.00000 -

FFPE 0.00026 1.00000 0.99296

**PedBE**

Friedman chi-squared = 22.68, df = 3, p-value = 4.708e-05

Pairwise comparisons using Wilcoxon rank sum exact test

CH F FF

F 6.5e-05 - -

FF 6.5e-05 1.00 -

FFPE 6.5e-05 0.74 0.38

**Wu**

Friedman chi-squared = 25.68, df = 3, p-value = 1.113e-05

Pairwise comparisons using Wilcoxon rank sum exact test

CH F FF

F 6.5e-05 - -

FF 6.5e-05 1.00 -

FFPE 6.5e-05 0.86 0.14

**TL**

Friedman chi-squared = 18.84, df = 3, p-value = 0.000295

Pairwise comparisons using Wilcoxon rank sum exact test

CH F FF

F 6.5e-05 - -

FF 6.5e-05 1 -

FFPE 6.5e-05 1 1

**BLUP**

Friedman chi-squared = 21.72, df = 3, p-value = 7.459e-05

Pairwise comparisons using Wilcoxon rank sum exact test

CH F FF

F 0.00195 - -

FF 0.00292 1.00000 -

FFPE 0.00078 1.00000 1.00000

**EN**

Friedman chi-squared = 16.92, df = 3, p-value = 0.000734

Pairwise comparisons using Wilcoxon rank sum exact test

CH F FF

F 0.017 - -

FF 0.023 1.000 -

FFPE 0.009 1.000 1.000

**3. Differences in DNAmAge of heart tissue stored under different conditions.**

**Horvath**

Friedman chi-squared = 5.4, df = 2, p-value = 0.06721

Pairwise comparisons using Wilcoxon rank sum exact test

F FF

FF 1.0 -

FFPE 0.5 0.5

**Hannum**

Friedman chi-squared = 2.4, df = 2, p-value = 0.3012

Pairwise comparisons using Wilcoxon rank sum exact test

F FF

FF 1 -

FFPE 1 1

**Levine**

Friedman chi-squared = 0.8, df = 2, p-value = 0.6703

Pairwise comparisons using Wilcoxon rank sum exact test

F FF

FF 1 -

FFPE 1 1

**SkinHorvath**

Friedman chi-squared = 3.8, df = 2, p-value = 0.1496

Pairwise comparisons using Wilcoxon rank sum exact test

F FF

FF 1.0 -

FFPE 1.0 0.5

**PedBE**

Friedman chi-squared = 7.8, df = 2, p-value = 0.02024

Pairwise comparisons using Wilcoxon rank sum exact test

F FF

FF 1.00 -

FFPE 0.37 0.19

**Wu**

Friedman chi-squared = 12.8, df = 2, p-value = 0.001662

Pairwise comparisons using Wilcoxon rank sum exact test

F FF

FF 1.00 -

FFPE 0.43 0.07

**TL**

Friedman chi-squared = 1.4, df = 2, p-value = 0.4966

Pairwise comparisons using Wilcoxon rank sum exact test

F FF

FF 1.00 -

FFPE 1.00 0.94

**BLUP**

Friedman chi-squared = 6.2, df = 2, p-value = 0.04505

Pairwise comparisons using Wilcoxon rank sum exact test

F FF

FF 1.00 -

FFPE 1.00 0.84

**EN**

Friedman chi-squared = 2.4, df = 2, p-value = 0.3012

Pairwise comparisons using Wilcoxon rank sum exact test

F FF

FF 1 -

FFPE 1 1
